# Pilot Study to Assess the Feasibility of a Mobile Unit for Remote Cognitive Screening of Isolated Elderly in Rural Areas

**DOI:** 10.3390/ijerph18116108

**Published:** 2021-06-05

**Authors:** Radia Zeghari, Rachid Guerchouche, Minh Tran Duc, François Bremond, Maria Pascale Lemoine, Vincent Bultingaire, Kai Langel, Zeger De Groote, Francis Kuhn, Emmanuelle Martin, Philippe Robert, Alexandra König

**Affiliations:** 1 Cognitive Behavior Technology (CoBTeK) Lab., FRIS-Université Côte d’Azur, 06100 Nice, France; rachid.guerchouche@inria.fr (R.G.); Francois.Bremond@inria.fr (F.B.); Philippe.ROBERT@univ-cotedazur.fr (P.R.); alexandra.konig@inria.fr (A.K.); 2 Institut National de Recherche en Informatique et en Automatique (INRIA), 06902 Valbonne, France; tran-duc.minh@inria.fr; 3 Centre Hospitalier Digne-les-Bains, 04995 Digne-Les-Bains, France; mplemoine@ch-digne.fr (M.P.L.); vbultingaire@ch-digne.fr (V.B.); 4 Janssen Clinical Innovation, 2340 Beerse, Belgium; KLangel1@its.jnj.com; 5 Modis, 2550 Kontich, Belgium; Zeger.De.Groote@modisbelgium.be; 6 Fédération d’Aide à Domicile en Milieu Rural (ADMR) des Alpes-de-Hautes-Provences, 04995 Digne-Les-Bains, France; f.kuhn06@gmail.com (F.K.); emartin@admr04.org (E.M.)

**Keywords:** telemedicine, cognitive assessment, videoconference, dementia, screening, mobile unit

## Abstract

Background: Given the current COVID-19 pandemic situation, now more than ever, remote solutions for assessing and monitoring individuals with cognitive impairment are urgently needed. Older adults in particular, living in isolated rural areas or so-called ‘medical deserts’, are facing major difficulties in getting access to diagnosis and care. Telemedical approaches to assessments are promising and seem well accepted, reducing the burden of bringing patients to specialized clinics. However, many older adults are not yet adequately equipped to allow for proper implementation of this technology. A potential solution could be a mobile unit in the form of a van, equipped with the telemedical system which comes to the patients’ home. The aim of this proof-of-concept study is to evaluate the feasibility and reliability of such mobile unit settings for remote cognitive testing. Methods and analysis: eight participants (aged between 69 and 86 years old) from the city of Digne-Les-Bains volunteered for this study. A basic neuropsychological assessment, including a short clinical interview, is administered in two conditions, by telemedicine in a mobile clinic (equipped van) at a participants’ home and face to face in a specialized clinic. The administration procedure order is randomized, and the results are compared with each other. Acceptability and user experience are assessed among participants and clinicians in a qualitative and quantitative manner. Measurements of stress indicators were collected for comparison. Results: The analysis revealed no significant differences in test results between the two administration procedures. Participants were, overall, very satisfied with the mobile clinic experience and found the use of the telemedical system relatively easy. Conclusion: A mobile unit equipped with a telemedical service could represent a solution for remote cognitive testing overcoming barriers in rural areas to access specialized diagnosis and care.

## 1. Introduction

With the rise of the COVID-19 pandemic crisis, now more than ever, remote solutions such as telemedicine platforms are of great importance to provide older people in isolated and underserved areas with access to specialized care services [1]. Additionally, the increasing risks caused by the social isolation of older people in these areas must be addressed rapidly.

In France, “medical desert” is a term used to describe regions where the population has inadequate access to healthcare [2]. In 2018, medical deserts continued to expand, with nearly 3.8 million French people living in an area under-provided with general practitioners (5.7% of the population), compared to 2.5 million (3.8% of the population) four years earlier [3]. Accessibility to general practitioners—located less than 20 min by car from the patients’ place of residence—decreased by 3.3% between 2015 and 2018. In total, France has 244 territories where general practitioners are under-provided, 1618 moderately provided and 961 well-provided. The French National Council of the Order of Physicians gives an overview of the situation in France with statistics related to the number of doctors per 10,000 inhabitants in 2019 (https://www.conseil-national.medecin.fr/ accessed on 23 March 2021).

These statistics represent the general healthcare access, and thus, mainly general practitioners. However, the situation is worse when it comes to specialized care. In France, in case of Alzheimer’s disease (AD), projections are alarming. In 2015, the number of people aged over 75 years old is estimated to 6.1 million. By 2030, this number is estimated to double (>12.2 million). In total, 900,000 people are affected by AD (two out of three are women), with 225,000 new cases each year. A total of 3 million people are estimated to be directly affected. This number will double by 2050 [4]. Today AD patients are underdiagnosed, with only around 50% of patients being diagnosed early, due to the restricted access to specialized medical professionals (e.g., psychiatrists, geriatricians, neurologists) [5]. As a result, the social and economic costs of the disease will increase, unless curative or preventive measures are quickly established. The most important rise in cases will be in countries with low and medium industrialization [6]. This is partly due to an increase in life expectancy, as well as a lack of an adequate prevention policy.

Since early detection of cognitive decline is a key factor when it comes to treating AD, facilitating access to experts is one solution to reduce the number of affected people. Currently, to get an AD assessment, patients have to undergo a long, burdensome process in specialty care centers where biological and cognitive evaluations are performed face to face using traditional pen and papers tests by a trained clinician. This can be very stressful for patients, especially those who have to travel far and those at early stages of cognitive impairment [7]. Additionally, the most frequently used general screening tests may not always be sensitive enough for identifying subtle changes in people at very early stages of dementia [8]. For this, more in-depth neuropsychological test measures are required. Most of these tests, however, could be empowered by technology and potentially be administered in a more standardized manner even remotely.

Over the past years, several technologies have been deployed in the field of dementia research using computerized cognitive testing, sensors, automatic speech, or image analysis for more objective and standardized evaluation of patients cognitive, behavioral, and emotional status [9]. These technologies allow to easily track the progress of symptoms. Furthermore, several studies have investigated the use of videoconferencing (VC) for remote cognitive assessments of dementia disorders [10,11], with the result that the measures obtained from virtual and face-to-face visits are highly comparable, and thus, reliable across modalities [12]. A longitudinal study noted differences between in-person assessment and VC scores only among the patients with severe impairments, which might be caused by difficulties in handling the technology. When it comes to establishing a clinical diagnosis of dementia, high levels of agreement were identified for a diagnosis made through VC with those made in person [13,14]. The use of VC is gradually increasing in diverse patient care settings, including primary care, critical care, neurology, behavioral health, psychiatry, and among other specialty areas [15]. Acceptability evaluations show that this form of assessment is well tolerated by the users [16]. Due to the COVID-19 pandemic, older people are increasingly even preferring virtual visits as they are of particular risk [1], making them no longer a need exclusively for those in rural areas.

Cognitive assessments are very challenging to perform virtually, as it requires standardized administration of visual stimuli; hence, translating tests across modalities is a cumbersome but necessary process. Until now, most research in this field examined the use of common VC platforms such as ©Skype or Zoom for remote testing. These platforms have security and practical limitations, pointing to a need for more adapted solutions. Studies found that verbally mediated tasks, such as learning lists, digit span or verbal fluency, were not affected by remote assessment [17]. Critical are tests relying on visual materials and those involving motor functions, physical contact (e.g., prehension behavior), or tests that require drawing on paper (e.g., visuospatial tests). The latter issue can be solved by using tactile tablets for VC systems that include this feature.

**Figure 1:**
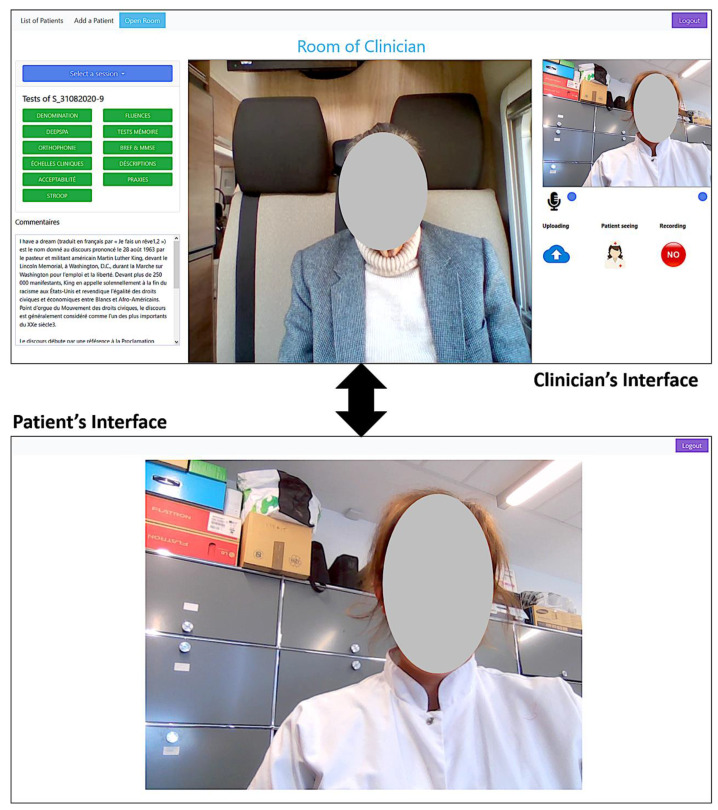
View of both the clinician’s and patient’s interfaces. The clinician’s interface allows several capabilities of controlling and sharing visual content (images and texts). The patient’s interface is simplified to make it easy to use for older people not used to computers. For some clinical tests, the possibility is given to the participant, when they are used to computers, to interact with the system (click on buttons, check boxes, or select images).

For this, a VC tool was developed (Figure 1) and specifically designed to perform remotely a full range of neuropsychological tests. In addition to already existing VC tools, it has two different adapted interfaces. The first one is for the clinician (left image), with all tests and clinical scales implemented and available on a platform with visual content, task-specific timers, and an automatic scoring system. The second one is for the patient (right image), and is very simplified and mainly shows the clinician or the test content. For each test, speech and video can be recorded for later post-processing and analysis.

Most studies have focused on providing services to rural patients via VC from an expert medical center to a rural clinic, which still implied that patients needed to travel. However, providing services directly to a patients’ home increases the challenges related to internet connection, camera quality, or privacy, but would represent, in response to the current COVID-19 pandemic, a valuable solution to the ongoing need to continue diagnosing AD. Therefore, the idea of implementing mobile units (MU) for virtual memory consultations has been suggested, since similar approaches were successfully tested in other diseases [18,19,20]. Bringing a mobile ‘clinic’ equipped with VC to the patients’ home would allow them to overcome certain technology-related barriers and ensure a controlled testing environment by allowing the patient to stay comfortably at home. One study in the US reported on a similar pilot project providing access to healthcare services to children through a telemedical service connected to a mobile clinic [21]. MU have served to extend access to early cancer screening in numerous countries [20] with encouraging outcomes.

Therefore, the overall aim of this first of its kind study is to evaluate the feasibility of using an MU to reach isolated older people for administering remotely cognitive assessments. The study targets to, firstly, compare results from VC administration of a short screening cognitive tests to the classical face-to-face administration, and secondly, assess acceptability among the users, participants, care providers, and clinicians.

## 2. Materials and Methods

### 2.1. Equipment

**Figure 2:**
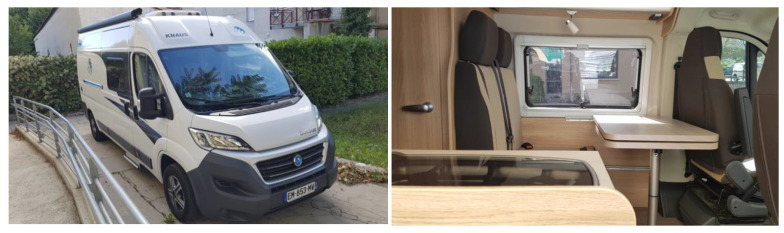
Exterior and Interior view of the camper van.

The materials:

The van: We rented a camper van that had a comfortable seat with a table and electric outlets (see Figure 2).

**Figure 3:**
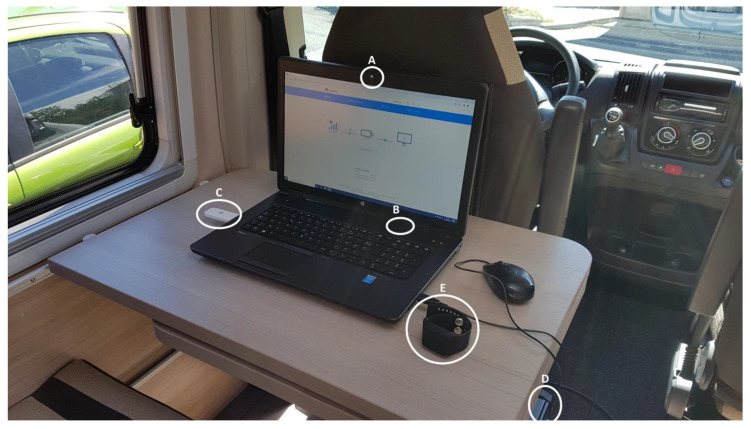
The laptop from the participants’ side (**A**) integrated microphone and webcam. (**B**) integrated speakers. (**C**) 4G key for internet connection. (**D**) Converter battery attached to the van’s electric plug to charge the laptop. (**E**) Empatica E4 wristband for stress measurement retrieval.

Computer: we used a 17-inch HP Zbook laptop, which already had an embedded webcam, a microphone, and speakers. The 17-inch screen allowed comfortable communication and enough space to display tests’ contents (see Figure 3). The laptop was powered by its internal battery during the tests and was charged when needed using the van’s electric plugs.

Internet connection: in order to ensure internet connection, we used a 4G key with unlimited data subscription (see Figure 3).

Stress measurements: in order to measure the stress level and acquire physiological data during the assessments (face-to-face and remote), we used the Empatica E4 wristband. The E4 is a medical-grade wearable device that offers real-time physiological data acquisition, enabling researchers to conduct in-depth analysis and visualization. The E4 combines several sensors:

- PPG Sensor: Measures Blood Volume Pulse (BVP), from which heart rate variability can be derived.
- Three-axis Accelerometer: Captures motion-based activity.
- EDA Sensor (GSR Sensor): Measures the constantly fluctuating changes in certain electrical properties of the skin.
- Infrared Thermopile: Reads peripheral skin temperature.

The E4 wristband was worn by the participants just before starting the assessments (see Figure 3).

### 2.2. The Video Conference (VC) System

The developed VC tool is a web-based platform fully dedicated to diagnosing, screening, and monitoring of cognitive disorders. This tool was developed using the latest advances in information and communication technologies to provide remote care through a web platform using any internet browser. This web platform allows an easy and direct connection between a clinician (e.g., neuropsychologist) and their patient or study participant. Both connect to the web platform using their respective identifiers and passwords. The patient’s interface is designed in a simple way that allows its use by individuals who are not familiar or not able to use computers and internet browsing. The clinician’s interface is more elaborated to allow several functionalities (see Figure 1).

The platform offers the following capabilities:

- Clinical interviewing with the patient
  ○ POption to video and/or audio record the conversation
- Performing the clinical tests:
  ○ More than 20 known and widely used neuropsychological tests and clinical scales are already implemented and validated (in French) such as: Mini Mental State Examination (MMSE) ([22]), Free Cued Selective Recall Task (FCSRT), such as the RL/RI 16 items [23] and the five-word screening test” test [24], Semantic and Phonemic verbal fluencies, denomination tasks, description tasks, and neuropsychiatric scales, such as the Geriatric Depression Scale [25] and Apathy Inventory [26].
  ○ Content sharing with the study participant: image, video, and text.
  ○ Reporting and saving test scores.
  ○ Video and/or audio recording of the test session (patient side).
- During a clinical test:
  ○ The patient can remain passive and does not have to interact with the system (everything is done with verbal communication).
  ○ The patient can interact with the system if they are comfortable with the technology: click on buttons, check boxes, or select an image.

As most studies until now used publicly available VC software (e.g., Skype, Zoom, etc.) to perform tests, this platform has the advantage that it is fully and exclusively designed to perform remote cognitive assessments with all the capabilities of displaying the specific content of each test. Assessments are made in standardized conditions, and the scoring system (semi-automatic) allows to store the data (including recorded videos/audio) on secured servers. The platform allows the clinician to review the test results and to re-listen (or re-watch) speech (or video) files of any test in case of doubt or for additional decision making, even after the end of the assessment.

Finally, this platform is currently used as a research tool in order to investigate the possibility of extracting additional visual and speech biomarkers from the recorded video and audio files. For this, artificial intelligence and machine learning techniques are applied to analyze body language (facial expressions, gaze and head directions, gestures, etc.) and speech (tonalities, repetitions, silence, etc.). These analyses aim at providing medical professionals with extra information about patients’ cognitive, emotional, and functional state.

### 2.3. Participants

For this observational cross-over study, we included, in the first round, 10 participants that were referred by a local association providing help to isolated older people, the ADMR (Aide à domicile en milieu rural/Home services in rural areas). We further aimed to include 20 older adults in total from the region of Digne-les-Bains, France over an inclusion period of 12 months. The inclusion criteria: subjects living in the region of Digne-les-Bains; over 55 years old; and native French speakers. Exclusion criteria: significant vision and auditory problems which would impact ability to perceive and understand the clinician. Written, informed consent was obtained from all subjects.

### 2.4. Procedure/Protocol

A brief neuropsychological assessment consisting of a short clinical interview followed by a set of cognitive screening tests was administered face to face and via the VC system in the MU two weeks apart by two different psychologists. Finally, all subjects filled out an acceptability scale after each condition. We designed a cross-over procedure so that half of the participants experienced the face-to-face screening test first and the other half the videoconference screening test first. Two versions of the tests were administered to avoid learning effects for the three recall words of the MMSE, the five-words test (“Musée” and “Mimosa” lists), and Fluency tasks (semantic category: Fruits/Animals; letter: P/R). After the inclusion of all participants, the results obtained at the VC administration were compared to the classical face-to-face method to evaluate their reliability. Evaluation reports of the neuropsychological assessments obtained in this study will be sent to the referring clinicians of the hospital in Digne-Les-Bains. Participants and neuropsychologists of the study were asked to complete a questionnaire on their experience and acceptability of the VC-administered assessment (compared to the classical) and usage of the MU.

### 2.5. Data Collection and Availability

Data were collected at the Hospital Center in Digne-les-Bains; the assessments were performed remotely and face to face by clinicians from the Memory Clinic in Nice. For remote assessments, the VC software recorded scores, videos, and speech and stored them on a dedicated secured server, complying with healthcare data hosting regulations.

Highly sensitive data were collected, and only processed data can be obtained from a third party and will not be made publicly available.

### 2.6. Acceptability Evaluation

All participants were asked to answer a questionnaire (see supplementary file) on the acceptance of the videoconference as well as of the face-to-face modality for cognitive testing, including seven questions with a response ranging from 1 to 7, where 1 = I strongly disagree and 7 = I strongly agree. This questionnaire is based on the ‘System Usability Scale’ [27] and assesses the user experience, including an overall evaluation, if participants are satisfied, if they want to repeat the experience, their subjective stress level, attitudes and clarity of instructions as well as what type of method is preferred and why, and what could be improved. Each subject completed an acceptability scale after each evaluation.

**Figure 4:**
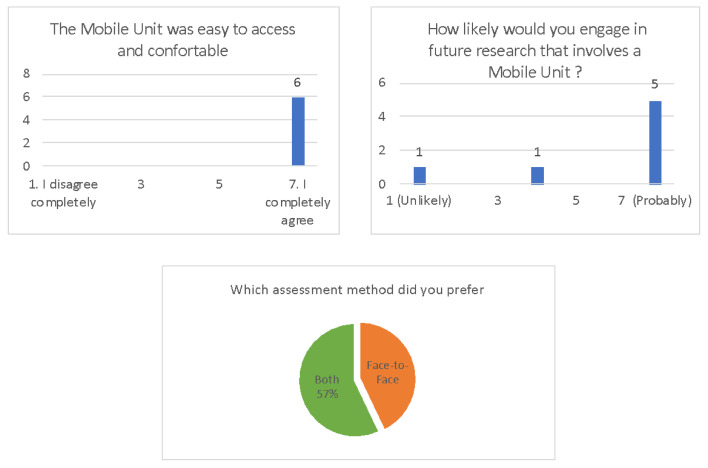
Results from the acceptability scales of the Mobile Unit condition.

The scales were adapted depending on the type of assessment (Face-to-face or Mobile Unit) and if it was the first or the second assessment. The mobile unit scale comprised two more open questions: “Was the Mobile Unit easy to access and comfortable?” and “How likely would you engage in future research that involves a Mobile Unit?”. The second assessment comprised a question to assess which method they preferred (see Figure 4).

### 2.7. Data Analysis

To assess the agreement between the two methods, we performed an Intraclass Correlation Coefficient analysis using SPSS Statistics version 23.0.0 for Mac software (Chicago, IL, USA). We compared the total scores of the MMSE (from 0 to 30) and the FAB (from 0 to 14). For the five words, we compared the total recall score (0–10), the total free recall score (from 0–10), and the delayed recall score (0–5). For verbal fluencies, we compared the total number of words said for the semantic and phonemic verbal fluencies. We compared, in the digit span tests, the greatest number series recalled both forwards and backwards. Due to our sample’s small size, we conducted a non-parametric Wilcoxon paired sample test for a group comparison. Stress measurements collected from the wearable device were not analyzed yet with the current sample.

## 3. Results

Ten subjects were included in the mobile unit setting’s first round. Two subjects withdrew from the study and did not attend the second appointment. We present here the results of four females and four males (Mean age: 76.5 (±6.12 yo) aged between 69 and 86 years old (see Table 1).

In Table 2, the cognitive tests’ mean scores collected with the different administration methods are presented. The means across the groups were highly similar, except for verbal fluencies.

To examine agreement of subject performances across both test conditions, we used Intraclass Correlations Coefficients (ICC) (see Table 3). MMSE, PVF, and DS onwards scores had significant agreement between the two methods. The FAB, 5 words—total recall score, SVF, and DS Backwards did not have significant agreement. The Wilcoxon Paired sample test did not show significant differences between the two methods.

### Acceptability Surveys

Participants. Results of the survey are presented in Table 4. Overall, participants seemed to score similarly for both methods, with even a slight preference for the MU administration. On item 5 (how likely they would recommend it to someone else), the MU obtained a much higher score than the face-to-face procedure. Stress level was, in return, higher in participants during the mobile unit. The additional questions show that most participants were willing to repeat this experience in the future and that they found the experience rather comfortable.

**Figure 5:**
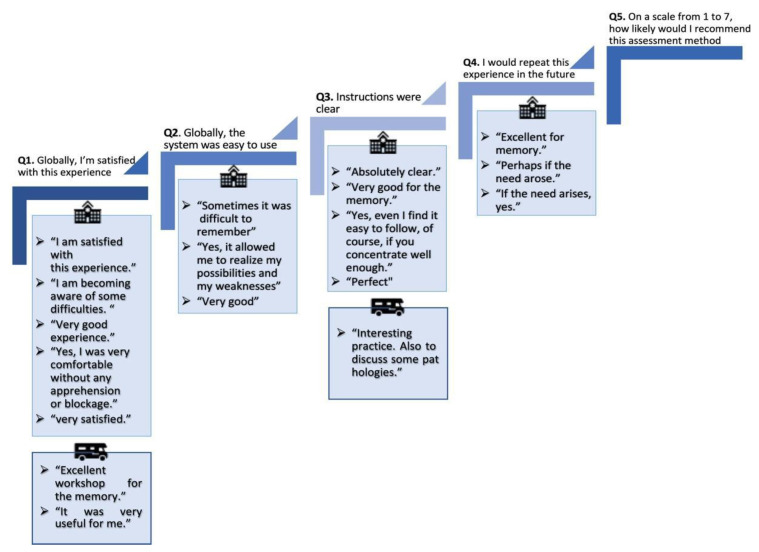
Participant feedback after each assessment.

We present in Figure 5 answers from acceptability questions specific to the MU condition and one question related to the preferred assessment method. Overall, six participants answered the question “The MU was easy to access and comfortable” and all answered, “I completely agree.” Five participants answered that they would “completely agree” to engage in future research that involves an MU, while one answered “unlikely” and one participant did not agree or disagree with the question. Of all seven subjects that answered the preferred method of assessment question, four chose both methods and three only the face-to-face method.

The acceptability scale comprised an open comment section after each question. All comments are displayed in Figure 5. Only three comments were made after the MU assessment opposed to 15 after the face-to-face assessment.

Stress measurements: We measured the electrodermal activity and heart rate variability to compare the stress level across both conditions more objectively and implicitly. The results are currently being processed and still need a larger sample for meaningful interpretation.

Care assistants: Feedback was gathered in addition to the persons involved in the study on their perception of how the participants experienced the MU assessment. Care assistants (from the ADMR) were positively surprised about the feasibility of implementing such a procedure to reach isolated older people and establish first contact with clinical specialists. Most comments were linked to the face-to-face condition, in which subjects had to be transported from different villages/towns to the hospital, which was long, tedious, and stressful. Neither of the two reported differences on subjects’ oral feedback between the conditions. One of the two care assistants commented on how easy and practical it was to move the van from one of the houses to a close parking lot with better internet access.

Technical/Organizational observations:

- At the beginning of the study, next to the assistant, an engineer was present to make sure that the technical equipment was used properly. However, since it was so easy to use, for the rest of study, no further assistance was required.
- In the MU, the laptop’s built-in camera had a narrow angle, which limited the view of the participants’ upper body.
- As these areas can have limited internet access, the VC system was not able to video record one subject during some tests. The system was designed so that video streaming and connection to the VC system are prioritized over video recording.
- One of the participants’ house location did not allow internet access to 4G. The MU was then moved, with the participant in it, to the nearest village hall, allowing a 3G connection.

## 4. Discussion

### 4.1. Principal Findings

In this pilot and ongoing study, we developed a VC platform and deployed it with an MU to evaluate the feasibility of such a tool for remote cognitive assessment in rural areas. We adapted five widely used cognitive tests within the VC system that were administered remotely by neuropsychologists to the participants in the MU. Preliminary results show that a MU intended for remote cognitive assessment is feasible even under challenging conditions, such as an unstable internet connection. The remote cognitive assessment results did not differ significantly from the face-to-face condition.Agreement between the two methods was met for the following three tests: Mini Mental State Examination (MMSE), Phonemic Verbal Fluency (PVF), and Onwards Digital Span (DS). The largest differences were found in the Semantic Verbal Fluency (SVF) task and the Backwards DS test. Thus, a bigger sample is needed to further validate these first results. Overall, participants showed very comparable performances across both modalities. These results are in line with previous research demonstrating the validity of remote neuropsychological assessments performed via VC [17].

Acceptability scale scores showed that all subjects considered that the MU was easy to access and as comfortable as the Face-to-Face (FF) condition. Subjects were, overall, satisfied with their experience in both conditions. One challenging aspect was the population who tested the technology. Older people are generally not accustomed to technology and can suffer from social biases which can make them reluctant to use them. Menachemi and colleagues identified a role of vulnerability and confidence as factors for the use of telemedicine tools [28]. These aspects can make the technology more difficult to use and even more so for those suffering from cognitive or motor impairments. However, in our pilot study, subjects considered the VC-based assessment in the MU just as easy as the FF condition and the instructions just as clear. One of our concerns was that the MU condition could be more stressful, which was slightly the case. In fact, when asked about their stress level, subjects rated a higher score on the scale in the MU condition than the FF. However, most subjects were satisfied with the MU. Some subjects raised an interesting aspect that moving to the hospital was more stressful than having the assessment in front of their house because they did not have to worry about being late or to miss their appointment. Half of the subjects preferred the FF condition and the other half both conditions. None chose only the MU condition, which underlines how important it is to keep face-to-face interactions. The overall experience was very positive and the approach surprisingly easy to implement. The study participants were satisfied to be able to stay at home while having the service come to their doorstep. Moreover, care assistants from the ADMR who participated in the study were satisfied as well with the MU setup, since they did not have to move subjects to the hospital afterwards. They were surprised at how easy it was implemented. It is important to maintain strong relationships with local care assistant associations, as they are the closest to our main target population and can be involved in follow-up consultations if necessary.

### 4.2. Advantages and Challenges of a VC System within an MU

The current context of the COVID-19 pandemic necessitates rapid implementation of such innovative solutions for remote cognitive and behavioral assessment of people at risk for cognitive impairment [29]. Our pilot study addresses particularly this urgent need, proposing a potential alternative framework for bringing telemedical application to isolated older people.

Mobile memory clinics are a growing field of interests. Studies have shown the use and benefits of mobile screening units in underserved and rural areas for hypertension, vision, cholesterol, obesity screening, etc. [30]. However, combining VC assessment and an MU provides new perspectives with a new framework. General practitioners, neurologists, and psychologists can assess their patient remotely with the help of a trained care assistant/caregiver onsite with this approach.

Although these results are preliminary (only eight subjects were included), it enabled us to observe certain aspects of remote cognitive assessment in an MU. Internet access was our main concern, as these rural areas are not always well served. Our pilot study allowed us to consider even more MU for remote assessment, since they can be easily moved around the initial area to reach better connectivity. For the purpose of this pilot feasibility study, we only included a short cognitive assessment, but we can imagine performing in the future a broader range of tests covering all major cognitive functions as well as neuropsychiatric symptoms and physiological measures.

The MU provides a controlled and standardized environment in comparison to patients’ homes and prevents invading their personal space. It accommodates all technological material and internet access to perform the VC assessment with the help of a trained assistant onsite. The MU can travel to patients’ homes or their village, and even nursing homes, to assess patients without transporting them to the nearest expert clinic, which can sometimes be a long drive away, causing confusion, stress, and discomfort. The latter is especially important for disabled patients, who can suffer from these long drives. Thus, MU could serve in medical deserts for frontline dementia screening as well as in combination with other services for regular health checkups by allowing at same time to reduce social isolation and loneliness.

The presence of an assistant in the MU will allow patients to engage in human contact, and the assistant can facilitate the therapeutic alliance, assist in every technical aspect (connect the computer to the VC platform), and equip the patient with physiological sensors if needed. Thus, there is a need to involve local associations who work with older people, as they are already trained to support them and are well informed of their health situation. However, involving a care assistant to drive the vehicle, who has proper technical training to assist if there is any material dysfunction and a proper clinical training with this particular population, is an additional cost that needs to be considered.

Potential risks of this method could be data security, patient privacy, and confidentiality, as well as a reduced amount of clinical data that can be acquired and the eventual negative impact of the quality of patient–clinician interaction [29].

By testing this VC successfully in an MU with a population that is very unfamiliar with technology and can suffer from cognitive impairment, we can consider the benefits of applying it to other populations (e.g., young adults with neurological or psychiatric disorders, children) and other regions. Additionally, such remote assessment solutions would allow for isolated people to get access to participation in clinical trials, which has been, until now, mainly reserved for people living in urban areas close to specialized clinics.

### 4.3. Limitations

The main limitation of our study is the small sample size. Due to the COVID-19 pandemic, our second round of inclusions could not be carried out. Moreover, in this MU pilot, for practical reasons, we could only include a few tests and could not cover all cognitive functions. We did perform in parallel a larger study with the VC installed in a rural clinic with a more complete battery of cognitive tests as well as their alternative versions. Finally, all participants were recruited through the ADMR, which could have caused a selection bias, since they work specifically with older people with a certain loss of autonomy.

## 5. Conclusions

MU represents an untapped potential resource for healthcare systems, particularly during international emergencies such as the COVID-19 pandemic, allowing to reach underserved populations in isolated areas [31]. Such remote ‘hybrid’ solutions can overcome the barriers to getting access to health care services often experienced by isolated communities and establish a vital link with clinical experts and research facilities. Moreover, clinical trials could benefit from reaching potential participants in these areas. Reduction of the travel burden on patients and caregivers would largely facilitate enrollment. As gradually more clinicians consider offering services via telemedicine, it is important to underline that our VC system allows for a broad range of clinical services to be offered to patients.

## Figures and Tables

**Table 1:** Demographic characteristics.

		*n*	%		
Sex					
	Females	4	50		
	Males	4	50		
Education					
	Primary	2	25		
	Secondary	3	38		
	High	3	38		
Age, years		mean	sd	min	max
		76.7	6.12	69	86

**Table 2:** Group comparison of the cognitive assessments. Mean (Standard deviation).

N = 8		Mobile Unit		Face to Face	
Global Functioning	MMSE	27.38	(3.20)	26.88	(3.31)
Executive functions	FAB	12.50	(0.93)	12.63	(1.92)
Memory (5 words)	Total recall	9.88	(0.35)	9.88	(0.35)
	Free recall	8.13	(1.73)	8.88	(0.99)
	Delayed recall	4.88	(0.35)	4.88	(0.35)
Verbal Fluency	SVF	21.88	(11.04)	19.75	(2.60)
	PVF	20.25	(8.12)	18.00	(7.50)
Working memory (Digit Span)	Onwards	5.63	(1.51)	5.38	(1.30)
	Backwards	4.63	(1.19)	4.00	(0.76)

MMSE: Mental Mini State Examination; FAB: Frontal Assessment Battery; 5 words: 5 mots de Dubois; SVF: Semantic Verbal Fluency; PVF: Phonemic Verbal Fluency.

**Table 3:** Differences and agreement of scores between MU and FF assessment.

Tests	Wilcoxon Paired Sample	ICC		
	*p*-Value	Coefficient	Lower Bound	Upper Bound
MMSE	0.279	0.964 ***	0.835	0.993
FAB	0.863	0.297	−4.697	0.869
5 words—Total recall	1	−0.4	−33.787	0.758
SVF	0.674	0.117	−5.598	0.834
PVF	0.115	0.924 ***	0.630	0.985
DS Onwards	0.414	0.894 **	0.505	0.979
DS Backwards	0.236	0	−2.937	0.788

MMSE: Mental Mini State Examination; FAB: Frontal Assessment Battery; 5 words: 5 mots de Dubois; SVF: Semantic Verbal Fluency; PVF: Phonemic Verbal Fluency. ** *p* < 0.01; *** *p* < 0.001.

**Table 4:** Acceptability survey. Questions were rated from 1 “I completely disagree” to 7 “I completely agree”. N = 8. Mean (SD).

	Face-to-Face		Mobile Unit	
Q1. Globally, I’m satisfied with this experience	7.0	(0.0)	7.0	(0.0)
Q2. Globally, the system was easy to use	6.6	(1.1)	7.0	(0.0)
Q3. Instructions were clear	6.4	(1.5)	7.0	(0.0)
Q4. I would repeat this experience in the future	6.6	(1.1)	6.6	(1.1)
Q5. On a scale from 1 to 7, how likely would I recommend this assessment method?	6.7	(0.6)	7.2	(0.4)
Q6. Globally, my stress level was	4.8	(1.0)	5.6	(1.5)

## Data Availability

The data that support the findings of this study are available from the corresponding author upon reasonable request.

## Supplementary Materials

The following are available online at https://www.mdpi.com/article/10.3390/ijerph18116108/s1, Questionnaire.
