# Supplementary material for: Pilot Study to Assess the Feasibility of a Mobile Unit for Remote Cognitive Screening of Isolated Elderly in Rural Areas
Source: Int J Environ Res Public Health. 2021 Jun 5;18(11):6108. doi: 10.3390/ijerph18116108 (PMC8201036; doi:10.3390/ijerph18116108)
Supplement: Supplementary file 1 [file ijerph-18-06108-s001.zip › ijerph-1210552-supplementary.pdf]

# French User Experience assessment

Notes:
Version A/B : First/Second assessment
Face to Face/Mobile Unit: If the User Experience assessment was completed after the Face-to-Face or the Mobile Unit condition.

1 – Face-to-face Version A…………………………………..P2
2 – Face-to-Face Version B………………………………. P4
3 – Mobile Unit Version A………………………………P6
4 – Mobile Unit Version B………………………………P9

## Évaluation de l'expérience utilisateur

### Face à face - Version A (sans comparaison)

Questionnaire sur l'acceptation de la procédure à distance pour les tests cognitifs

Consigne : Merci de répondre à ce questionnaire. **Pour répondre, veuillez cocher la case correspondant à votre choix.**

1. Je suis globalement satisfait(e) de cette expérience.

Complètement en désaccord — Complètement d'accord

| 1 | 2 | 3 | 4 | 5 | 6 | 7 |
|---|---|---|---|---|---|---|

Commentaires :

______________________________________________________________________________

______________________________________________________________________________

______________________________________________________________________________

2. Globalement, le système est facile à utiliser.

Complètement en désaccord — Complètement d'accord

| 1 | 2 | 3 | 4 | 5 | 6 | 7 |
|---|---|---|---|---|---|---|

Commentaires :

______________________________________________________________________________

______________________________________________________________________________

______________________________________________________________________________

3. Les instructions étaient claires et compréhensibles.

Complètement en désaccord — Complètement d'accord

| 1 | 2 | 3 | 4 | 5 | 6 | 7 |
|---|---|---|---|---|---|---|

Commentaires :

______________________________________________________________________________

______________________________________________________________________________

______________________________________________________________________________

4. Je répéterais cette expérience dans le futur.

Complètement en désaccord — Complètement d'accord

| 1 | 2 | 3 | 4 | 5 | 6 | 7 |
|---|---|---|---|---|---|---|

Commentaires :

______________________________________________________________________________

______________________________________________________________________________________

______________________________________________________________________________________

5. Sur une échelle de 1 à 7, quelle est le degré de probabilité pour que vous recommandiez cette méthode d'évaluation à un ami ou un collègue ?

Complètement en désaccord — Complètement d'accord

| 1 | 2 | 3 | 4 | 5 | 6 | 7 |
|---|---|---|---|---|---|---|

Commentaires :

______________________________________________________________________________________

______________________________________________________________________________________

______________________________________________________________________________________

6. Sur une échelle de 1 à 7 : globalement, j'ai l'impression que mon niveau de stress pendant l'évaluation était...

Inférieur à d'habitude — Moyen — Supérieur à d'habitude

| 1 | 2 | 3 | 4 | 5 | 6 | 7 |
|---|---|---|---|---|---|---|

Commentaires :

______________________________________________________________________________________

______________________________________________________________________________________

______________________________________________________________________________________

7. Qu'est-ce qui manquait ou qui vous a déçu durant cette expérience ?

______________________________________________________________________________________

______________________________________________________________________________________

8. Qu'est-ce que vous avez le plus/le moins aimé dans cette procédure ?

______________________________________________________________________________________

______________________________________________________________________________________

9. Quelle(s) proposition(s) feriez-vous pour améliorer l'évaluation en face à face ?

______________________________________________________________________________________

_____________________________________________________________________________________

## Évaluation de l'expérience utilisateur

### Face à face - Version B (avec comparaison)

Questionnaire sur l'acceptation de la procédure à distance pour les tests cognitifs

Consigne : Merci de répondre à ce questionnaire. **Pour répondre, veuillez cocher la case correspondant à votre choix.**

1. Je suis globalement satisfait(e) de cette expérience.

Complètement en désaccord — Complètement d'accord

| 1 | 2 | 3 | 4 | 5 | 6 | 7 |
|---|---|---|---|---|---|---|

Commentaires :

______________________________________________________________________________

______________________________________________________________________________

______________________________________________________________________________

2. Globalement, le système est facile à utiliser.

Complètement en désaccord — Complètement d'accord

| 1 | 2 | 3 | 4 | 5 | 6 | 7 |
|---|---|---|---|---|---|---|

Commentaires :

______________________________________________________________________________

______________________________________________________________________________

______________________________________________________________________________

3. Les instructions étaient claires et compréhensibles.

Complètement en désaccord — Complètement d'accord

| 1 | 2 | 3 | 4 | 5 | 6 | 7 |
|---|---|---|---|---|---|---|

Commentaires :

______________________________________________________________________________

______________________________________________________________________________

______________________________________________________________________________

4. Je répéterais cette expérience dans le futur.

Complètement en désaccord — Complètement d'accord

| 1 | 2 | 3 | 4 | 5 | 6 | 7 |
|---|---|---|---|---|---|---|

Commentaires :

______________________________________________________________________________

______________________________________________________________________
______________________________________________________________________

5. Sur une échelle de 1 à 7, quelle est le degré de probabilité pour que vous recommandiez cette méthode d'évaluation à un ami ou un collègue ?

Complètement en désaccord Complètement d’accord

| 1 | 2 | 3 | 4 | 5 | 6 | 7 |
|---|---|---|---|---|---|---|

Commentaires :

______________________________________________________________________
______________________________________________________________________
______________________________________________________________________

6. Sur une échelle de 1 à 7 : globalement, j'ai l'impression que mon niveau de stress pendant l'évaluation était...

Inférieur à d’habitude Moyen Supérieur à d’habitude

| 1 | 2 | 3 | 4 | 5 | 6 | 7 |
|---|---|---|---|---|---|---|

Commentaires :

______________________________________________________________________
______________________________________________________________________
______________________________________________________________________

7. Quelle méthode d'évaluation préférez-vous, en face-à-face ou par vidéoconférence ?

| Face-à-face | Vidéoconférence |
|---|---|

8. Qu'est-ce qui manquait ou qui vous a déçu durant cette expérience ?

______________________________________________________________________
______________________________________________________________________

9. Qu'est-ce que vous avez le plus/le moins aimé dans cette procédure ?

______________________________________________________________________
______________________________________________________________________

10. Quelle(s) proposition(s) feriez-vous pour améliorer l'évaluation en face à face ?

______________________________________________________________________
______________________________________________________________________

## Évaluation de l'expérience utilisateur

**Unité Mobile – Version A (sans comparaison)**

Questionnaire sur l'acceptation de la procédure à distance pour les tests cognitifs

Consigne : Merci de répondre à ce questionnaire. **Pour répondre, veuillez cocher la case correspondant à votre choix.**

1. Je suis globalement satisfait(e) de cette expérience.

Complètement en désaccord Complètement d’accord

| 1 | 2 | 3 | 4 | 5 | 6 | 7 |
|---|---|---|---|---|---|---|

Commentaires :

_______________________________________________

_______________________________________________

_______________________________________________

2. Globalement, le système est facile à utiliser.

Complètement en désaccord Complètement d’accord

| 1 | 2 | 3 | 4 | 5 | 6 | 7 |
|---|---|---|---|---|---|---|

Commentaires :

_______________________________________________

_______________________________________________

_______________________________________________

3. Les instructions étaient claires et compréhensibles.

Complètement en désaccord Complètement d’accord

| 1 | 2 | 3 | 4 | 5 | 6 | 7 |
|---|---|---|---|---|---|---|

Commentaires :

_______________________________________________

_______________________________________________

_______________________________________________

4. Je répéterais cette expérience dans le futur.

Complètement en désaccord Complètement d’accord

| 1 | 2 | 3 | 4 | 5 | 6 | 7 |
|---|---|---|---|---|---|---|

Commentaires :

_______________________________________________

______________________________________________________________________________
______________________________________________________________________________

5. Sur une échelle de 1 à 7, quelle est le degré de probabilité pour que vous recommandiez cette méthode d'évaluation à un ami ou un collègue ?

Complètement en désaccord Complètement d'accord

| 1 | 2 | 3 | 4 | 5 | 6 | 7 |
|---|---|---|---|---|---|---|

Commentaires :
______________________________________________________________________________
______________________________________________________________________________
______________________________________________________________________________

6. Sur une échelle de 1 à 7 : globalement, j'ai l'impression que mon niveau de stress pendant l'évaluation était...

Inférieur à d'habitude Moyen Supérieur à d'habitude

| 1 | 2 | 3 | 4 | 5 | 6 | 7 |
|---|---|---|---|---|---|---|

Commentaires :
______________________________________________________________________________
______________________________________________________________________________
______________________________________________________________________________

7. L'unité mobile (la camionnette) était facile d'accès et confortable pour effectuer l'évaluation. [Veuillez motiver votre réponse dans la section des commentaires]

Complètement en désaccord Complètement d'accord

| 1 | 2 | 3 | 4 | 5 | 6 | 7 |
|---|---|---|---|---|---|---|

Commentaires :
______________________________________________________________________________
______________________________________________________________________________
______________________________________________________________________________

8. Sur une échelle de 1 à 7, quelle est la probabilité que vous souhaitiez vous engager dans de futures études en utilisant une unité mobile (camionnette) ?

Peu probable du tout Indécis Je participerais

| 1 | 2 | 3 | 4 | 5 | 6 | 7 |
|---|---|---|---|---|---|---|

Commentaires :
_______________________________________________________________________________
_______________________________________________________________________________
_______________________________________________________________________________

9. Qu'est-ce qui manquait ou qui vous a déçu durant cette expérience ?
_______________________________________________________________________________
_______________________________________________________________________________

10. Qu'est-ce que vous avez le plus/le moins aimé dans cette procédure ?
_______________________________________________________________________________
_______________________________________________________________________________

11. Quelle(s) proposition(s) feriez-vous pour améliorer l'évaluation en vidéoconférence ?
_______________________________________________________________________________
_______________________________________________________________________________

# Évaluation de l'expérience utilisateur

**Unité Mobile – Version B (avec comparaison)**

Questionnaire sur l'acceptation de la procédure à distance pour les tests cognitifs

Consigne : Merci de répondre à ce questionnaire. **Pour répondre, veuillez cocher la case correspondant à votre choix.**

1. Je suis globalement satisfait(e) de cette expérience.

Complètement en désaccord | Complètement d'accord

| 1 | 2 | 3 | 4 | 5 | 6 | 7 |
|---|---|---|---|---|---|---|

Commentaires :

______________________________________________________________________________
______________________________________________________________________________
______________________________________________________________________________

2. Globalement, le système est facile à utiliser.

Complètement en désaccord | Complètement d'accord

| 1 | 2 | 3 | 4 | 5 | 6 | 7 |
|---|---|---|---|---|---|---|

Commentaires :

______________________________________________________________________________
______________________________________________________________________________
______________________________________________________________________________

3. Les instructions étaient claires et compréhensibles.

Complètement en désaccord | Complètement d'accord

| 1 | 2 | 3 | 4 | 5 | 6 | 7 |
|---|---|---|---|---|---|---|

Commentaires :

______________________________________________________________________________
______________________________________________________________________________
______________________________________________________________________________

4. Je répéterais cette expérience dans le futur.

Complètement en désaccord | Complètement d'accord

| 1 | 2 | 3 | 4 | 5 | 6 | 7 |
|---|---|---|---|---|---|---|

Commentaires :

______________________________________________________________________________
______________________________________________________________________________
______________________________________________________________________________

5. Sur une échelle de 1 à 7, quelle est le degré de probabilité pour que vous recommandiez cette méthode d'évaluation à un ami ou un collègue ?

Complètement en désaccord Complètement d'accord

| 1 | 2 | 3 | 4 | 5 | 6 | 7 |
|---|---|---|---|---|---|---|

Commentaires :

______________________________________________________________________________
______________________________________________________________________________
______________________________________________________________________________

6. Sur une échelle de 1 à 7 : globalement, j'ai l'impression que mon niveau de stress pendant l'évaluation était...

Inférieur à d'habitude Moyen Supérieur à d'habitude

| 1 | 2 | 3 | 4 | 5 | 6 | 7 |
|---|---|---|---|---|---|---|

Commentaires :

______________________________________________________________________________
______________________________________________________________________________
______________________________________________________________________________

7. L'unité mobile (la camionnette) était facile d'accès et confortable pour effectuer l'évaluation. [Veuillez motiver votre réponse dans la section des commentaires]

Complètement en désaccord Complètement d'accord

| 1 | 2 | 3 | 4 | 5 | 6 | 7 |
|---|---|---|---|---|---|---|

Commentaires :

______________________________________________________________________________
______________________________________________________________________________
______________________________________________________________________________

8. Sur une échelle de 1 à 7, quelle est la probabilité pour que vous souhaitiez vous engager dans de futures études en utilisant une unité mobile (camionnette) ?

Peu probable Indécis Je participerais

| 1 | 2 | 3 | 4 | 5 | 6 | 7 |
|---|---|---|---|---|---|---|

Commentaires :

______________________________________________________________________________

______________________________________________________________________________

______________________________________________________________________________

9. Quelle méthode d'évaluation préférez-vous, en face-à-face ou par vidéoconférence ?

| **Face-à-face** | **Vidéoconférence** |
| --- | --- |

10. Qu'est-ce qui manquait ou qui vous a déçu durant cette expérience ?

______________________________________________________________________________

______________________________________________________________________________

11. Qu'est-ce que vous avez le plus/le moins aimé dans cette procédure ?

______________________________________________________________________________

______________________________________________________________________________

12. Quelle(s) proposition(s) feriez-vous pour améliorer l'évaluation en vidéoconférence ?

______________________________________________________________________________

______________________________________________________________________________
